# Discovery of Novel Reductive Elimination Pathway for 10-Hydroxywarfarin

**DOI:** 10.3389/fphar.2021.805133

**Published:** 2022-01-13

**Authors:** Dakota L. Pouncey, Dustyn A. Barnette, Riley W. Sinnott, Sarah J. Phillips, Noah R. Flynn, Howard P. Hendrickson, S. Joshua Swamidass, Grover P. Miller

**Affiliations:** ^1^ Department of Biochemistry and Molecular Biology, College of Medicine, University of Arkansas for Medical Sciences, Little Rock, AR, United States; ^2^ Department of Pharmaceutical Sciences, College of Pharmacy, University of Arkansas for Medical Sciences, Little Rock, AR, United States; ^3^ Department of Pathology and Immunology, Washington University School of Medicine, St. Louis, MO, United States; ^4^ Department of Pharmaceutical Social and Administrative Sciences, McWhorter School of Pharmacy, Samford University, Birmingham, AL, United States

**Keywords:** anticoagulation, warfarin, hydroxywarfarin, metabolism, modeling, reduction, CBR1, AKR1C3

## Abstract

Coumadin (R/S-warfarin) anticoagulant therapy is highly efficacious in preventing the formation of blood clots; however, significant inter-individual variations in response risks over or under dosing resulting in adverse bleeding events or ineffective therapy, respectively. Levels of pharmacologically active forms of the drug and metabolites depend on a diversity of metabolic pathways. Cytochromes P450 play a major role in oxidizing R- and S-warfarin to 6-, 7-, 8-, 10-, and 4′-hydroxywarfarin, and warfarin alcohols form through a minor metabolic pathway involving reduction at the C11 position. We hypothesized that due to structural similarities with warfarin, hydroxywarfarins undergo reduction, possibly impacting their pharmacological activity and elimination. We modeled reduction reactions and carried out experimental steady-state reactions with human liver cytosol for conversion of *rac*-6-, 7-, 8-, 4′-hydroxywarfarin and 10-hydroxywarfarin isomers to the corresponding alcohols. The modeling correctly predicted the more efficient reduction of 10-hydroxywarfarin over warfarin but not the order of the remaining hydroxywarfarins. Experimental studies did not indicate any clear trends in the reduction for *rac*-hydroxywarfarins or 10-hydroxywarfarin into alcohol 1 and 2. The collective findings indicated the location of the hydroxyl group significantly impacted reduction selectivity among the hydroxywarfarins, as well as the specificity for the resulting metabolites. Based on studies with R- and S-7-hydroxywarfarin, we predicted that all hydroxywarfarin reductions are enantioselective toward *R* substrates and enantiospecific for *S* alcohol metabolites. CBR1 and to a lesser extent AKR1C3 reductases are responsible for those reactions. Due to the inefficiency of reactions, only reduction of 10-hydroxywarfarin is likely to be important in clearance of the metabolite. This pathway for 10-hydroxywarfarin may have clinical relevance as well given its anticoagulant activity and capacity to inhibit *S*-warfarin metabolism.

## Introduction

Coumadin (*rac*-warfarin) is frequently prescribed in the prophylaxis of thromboembolism in the setting of atrial fibrillation, stroke, and many other hypercoagulable conditions. This anticoagulant effect of warfarin is derived from the inhibition of vitamin K oxidoreductase complex 1 (VKORC1), which is involved in the recycling of vitamin K during the maturation of coagulation factors (Ansell, et al., 2008). Despite its important role in anticoagulant therapy since the 1950s, warfarin remains underutilized due to its interindividual unpredictability in dose response and narrow therapeutic range. Patients have their own response to the medication routinely tested to optimize dosing and mitigate the possibility of hemorrhaging (over-dosing) or clotting (under-dosing); nonetheless, warfarin remains in the top ten drugs for hospitalizations due to side effects (Wysowski et al., 2007). A deeper understanding of the underlying causes of response variation may lead to improved management strategies for achieving and maintaining an optimal dose, while simultaneously minimizing possible adverse drug events.

**FIGURE 1:**
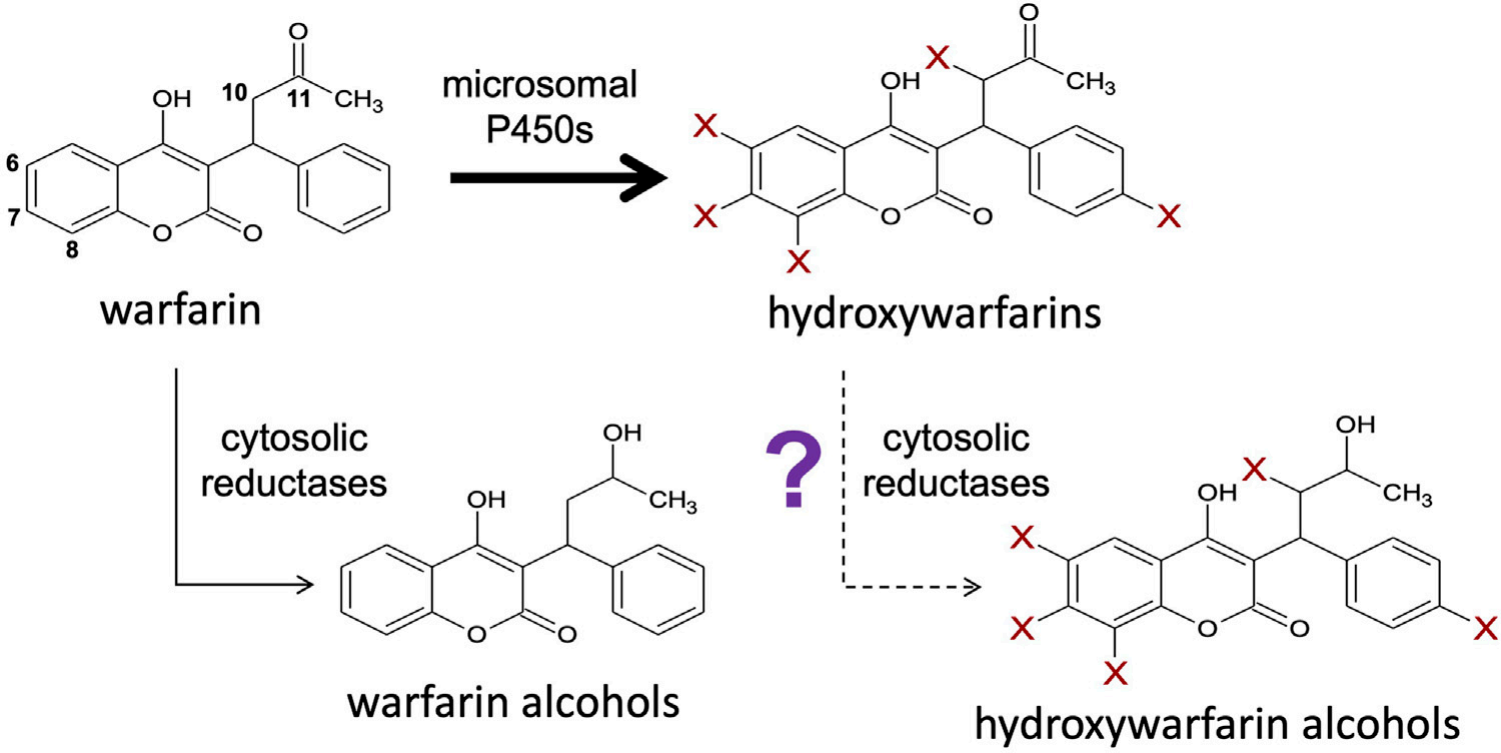
Hydroxywarfarins are the predominant metabolites of warfarin metabolism that may be cleared through a novel carbonyl reduction pathway. Warfarin is directly metabolized *via* a major pathway by membrane-bound cytochromes P450 to form hydroxywarfarins and a minor pathway by cytosolic reductases to form warfarin alcohols (Kaminsky and Zhang 1997; Jones and Miller 2011). Further reduction of the hydroxywarfarin major metabolites by reductases to form hydroxywarfarin alcohols may also be possible as a secondary reaction.

Warfarin therapy entails oral treatments of a racemic mixture of *R*- and *S*-warfarin. Reported R-warfarin levels in patients are on average 2-fold higher than *S*-warfarin, however *S-*warfarin is thought to be 3–5 times more effective as an anticoagulant than *R*-warfarin (Lewis et al., 1974; Jones et al., 2010a; Haque, et al., 2014; Miller 2010). In addition, warfarin potency is impacted by its extensive metabolism (Figure 1). Each warfarin enantiomer undergoes primarily oxidation into 6-, 7-, 8-, and 4′-hydroxywarfarins and a pair of 10-hydroxywarfarin isomers. Minor but relevant, competing pathways are warfarin reductions into pairs of alcohols (Kaminsky and Zhang 1997; Jones et al., 2010b; Jones and Miller 2011). Metabolism decreases levels of the parent drugs but do not necessarily eliminate the anticoagulant response (Lewis et al., 1973). Hydroxywarfarins and warfarin alcohols may still inhibit VKORC1 (Haque, et al., 2014) or even inhibit metabolism of the parent drugs (Jones, et al., 2010c) that may contribute to the overall anticoagulant effect. Those outcomes will ultimately depend on processes impacting warfarin metabolite levels. Warfarin hydroxylation introduces an essential site for glucuronidation leading to elimination of most hydroxywarfarins (Zielinska, et al., 2007; Bratton, et al., 2012; Kim, et al., 2019; Pugh, et al., 2018), as glucuronides in patient urine (Kaminsky and Zhang 1997; Miller, Jones, et al., 2009). 10-Hydroxywarfarin is the only primary metabolite that does not undergo glucuronidation and interestingly, is not eliminated in the urine as an unmodified metabolite, so that its elimination pathway remains unknown.

As an alternative to glucuronidation, we hypothesized that hydroxywarfarin reduction is an alternate, competing pathway for elimination of these primary metabolites. For each enantiomer, this process involves reduction of the C11 carbonyl into two possible alcohols or four for a racemic substrate mixture. Historically, studies carried out with *rac*-warfarin yielded partially resolvable isomers called alcohol 1 (minor metabolite) and alcohol 2 (major metabolite) (Alshogran, et al., 2014; Malátkova, et al., 2016). Our previous study further assessed the role of chirality in warfarin reductive reactions with authentic standards for all isomers (Barnette, et al., 2017). We determined that alcohol 1 consisted of two co-eluted alcohol isomers sharing common chiral orientations (*9R-11R*-hydroxywarfarin and *9S-11S*-hydroxywarfarin), whereas alcohol 2 was the combination of alcohols with mixed chiral centers (*9R-11S*-hydroxywarfarin and *9S-11R*-hydroxywarfarin). Nevertheless, *R*-warfarin was more efficiently metabolized than *S*-warfarin and both reactions resulted mainly in formation of the *S* alcohol by CBR1 and AKR1C3 (Malátkova, et al., 2016; Barnette, et al., 2017). Consequently, *rac*-warfarin reactions mainly yielded *9S-11S*-hydroxywarfarin (alcohol 1, minor metabolite), and *9R-11S*-hydroxywarfarin (alcohol 2, major metabolite). Given access only to *rac*-hydroxywarfarins, we anticipated similar metabolic profiles of diastereomeric isomers from reductive reactions. How the regiospecificity of warfarin hydroxylation would impact those subsequent reductive reactions remained unknown.

We tested then the hypothesized reduction of hydroxywarfarins through a combination of computational and experimental *in vitro* metabolic studies. For a rapid initial analysis, we predicted the likelihood for warfarin and hydroxywarfarin reduction at the C11 carbonyl using the publicly available Rainbow Model for metabolism developed by our group (Dang, et al., 2020). The model does not consider chirality but does yield a useful, scalable output for ranking the likelihood of reactions like reductions to occur. For experimental studies, we leveraged our previous methodologies (Barnette, et al., 2017) for characterizing R- and S-warfarin reduction by human liver cytosolic fractions to assess this metabolic pathway for 6-, 7-, 8-, 10-, and 4′-hydroxywarfarins. Isomeric mixtures were necessary for these studies given the lack of commercial availability of the individual isomers. Initially, we screened for metabolic activities and characterized the resulting metabolites by liquid chromatography coupled to mass spectrometry (LC-MS) to confirm the occurrence of reductions. We then determined conditions suitable for steady-state studies and measured reaction kinetics for *rac*-6-, 7-, 8-, and 4′-hydroxywarfarins along with the 10-hydroxywarfarin mix of isomers. We then chromatographically purified 7-hydroxywarfarin enantiomers and carried out steady-state studies to explore the impact of chirality toward reductase selectivity and specificity during reactions. It was not possible to carry out similar studies with 10-hydroxywarfarin due to the presence of chiral centers at positions 9 and 10 (Jones and Miller 2011) leading to four unresolvable isomers. 7- and 10-Hydroxywarfarin are metabolites of major metabolic pathways, and so we identified possible reductases responsible for their reduction using inhibitor phenotyping. Taken together, this study provides insights into the importance of hydroxywarfarin reduction *in vitro* and serves as a foundation to further evaluate the potential impact of this process on maintaining levels of active metabolites on the anticoagulant response in patients.

## Materials and Methods

### Materials

Unless otherwise stated, common reagents were ACS grade and purchased from Sigma-Aldrich. Toronto Research Chemicals was the source for *rac-*warfarin and *rac-*6-, 7-, 8-, and 4′-hydroxywarfarin along with the 10-hydroxywarfarin mixed isomers. High Performance liquid chromatography grade methanol and acetonitrile were obtained from Thermo Fisher (Pittsburgh, PA). Human liver cytosol pooled from 150 donors (HLC150) was purchased from Corning Gentest (Corning, NY).

### Modeling Hydroxywarfarin Reductions

For rapid insights on possible reactions, we predicted hydroxywarfarin reductions at the C11 carbonyl using our deep neural Rainbow Model that simultaneously labels sites of metabolism and classifies them into five key reaction classes: stable and unstable oxidations, dehydrogenation, hydrolysis, and reduction (Dang, et al., 2020). The model was trained on 9674 unique molecules and 20736 human *in vitro* and *in vivo* records, including 1590 reduction reactions, from literature-curated databases. This powerful tool is available for use free and online through our secure server at http://swami.wustl.edu/xenosite/p/phase1_rainbow. For each atom or bond site on the molecule, the model generates a score from 0 to 1.0, with higher values corresponding to greater likelihood for a reaction to occur at that location. This model output is analogous to a probability and provides a strategy to scale reaction relative likelihoods. In our case, we used this metric to rank hydroxywarfarin reductions in comparison to parent drug warfarin. We could then compare the findings to those from experimental kinetic studies, which are more labor and resource intensive.

### Steady-State Reduction of Warfarin and Hydroxywarfarins by Human Liver Cytosol

Steady-state studies involved a two-stage approach to first identify suitable reaction conditions and then to use them for determining kinetic profiles for reactions. Initially, control experiments were conducted to determine the optimal reaction time and protein concentration to ensure steady-state conditions. Hydroxywarfarin stocks were prepared in methanol solutions. For steady-state kinetic reactions, hydroxywarfarin aliquots were dried down using an evaporator under nitrogen using an Organomation Microvap Nitrogen Evaporator System (Organomation Associates, Inc., Berlin MA) to remove the carrier solvent. The residual *rac*-hydroxywarfarin was then resuspended in 50 mM potassium phosphate buffer pH 7.4 and sonicated for 5 min. HLC150 was added to the substrate to final concentrations of 0.25–1.0 mg/ml protein. The mixture was incubated for 10 min at 37°C with 350 rpm rotation and then the reaction was initiated with the addition of 1 mM NADPH. Reactions were quenched after 10, 20, 40, or 60 min using ice cold acetonitrile containing 1 µM coumatetryl (final) as an internal standard. Quenched reactions were centrifuged at 5°C at 2,500 rpm for 15 min with the supernatant transferred to a 96 well half-area plate for injection. Those studies identified conditions leading to linear response as a function of time and protein concentration, 40 min and 1.0 mg/ml protein, respectively, that were used in subsequent steady-state kinetic studies. Initial rates were measured for substrate concentrations ranging from 50 to 1,000 µM. Hydroxywarfarin alcohol initial rates were plotted as a function of substrate concentration and analyzed by comparing the fit of the data to the Michaelis-Menten equation (hyperbolic curve) and Hill equation (nonhyperbolic curve) using GraphPad Prism 9.2 (San Diego, CA). The best fit was determined using extra sum-of-squares F test.

### Characterization of Hydroxywarfarin Metabolites by Mass Spectrometry

For structural characterization, sample reactions were injected onto a Shimadzu UHFLC equipped with an SPD-10A UV–Vis (280 nm) and RF-10AXL fluorescence (excitation 280 nm, emission 650 nm) detectors.

Waters Acquity LC series (Waters-Millipore Corp, Milford, MA) and analytical separation were achieved on a 2.1 × 50 mm Hypersil Gold 5 µm column (Thermo Scientific). Parent compounds and metabolites were separated using a linear binary gradient (mobile phase A: water containing 0.1% formic acid, Mobile phase B: acetonitrile containing 0.1% formic acid). The flow rate was 0.4 ml/min, and the gradient was: initial 20% (B), 0–2.0 min 20–40% (B), 2.0–2.75 min 40 to 55% (B), 2.75–3.5 min 55–65% (B), held, and then returned to 20% (B) at 3.6 min. The total run time was 5.0 min. Subsequent mass spectrometry involved a Quattro Premier triple quadrupole mass spectrometer (Waters-Millipore Corp.), which used electrospray ionization to interface the LC system. Positive ions were generated using a cone voltage of 25 V. Product ions were generated using argon collision induced disassociation at a collision energy of 15 eV while maintaining a collision cell pressure of 1.2 × 10^−3^ torr. Detection was achieved using MS scan from 100 to 400 m*/z* for fragments of parent ions, *m/z* 311.2 for warfarin alcohols, and *m/*z 327.2 for hydroxywarfarin alcohols.

### Quantitative Analysis of Hydroxywarfarin Alcohol Metabolites

Samples were injected onto a 4.6 × 150 mm Zorbax Eclipse 3.5 µm XDB-C18 column (Agilent) heated to 45°C using a Shimadzu UHFLC equipped with SPD-10A UV–Vis and RF-10AXL fluorescence detectors. Analytes including warfarin, hydroxywarfarins, reduced metabolites, and coumatetryl (internal standard) were resolved with an isocratic method of 25 mM 4-(2-hydroxyethyl)-1-piperazineethanesulfonic acid (HEPES) buffer pH 6.5 and 3:1 methanol:acetonitrile. The elution of analytes was monitored by absorbance at 325 nm and fluorescence (excitation: 325 nm, emission: 393 nm). Peak areas were normalized to internal standard and quantified by inference using the substrate response. Due to the absence of authentic metabolite standards, we inferred their quantitation based on the corresponding responses. Molecular fluorescence was more sensitive than absorbance; however, carbonyl reduction impacted fluorescence but not absorbance based on initial studies with warfarin and warfarin alcohols. Consequently, for substrate and metabolite in each reaction, we calculated the response ratio as the normalized peak area for the fluorescence response divided by that for absorbance (R_flu/abs_). Division of the metabolite response ratio by that for substrate yielded a conversion factor, i.e., CF = Metabolite R_flu/abs_ ÷ Substate R_flu/abs_. Metabolite fluorescence responses could then be multiplied by the conversion factor to generate a response comparable to that for substrate making its quantitation by inference using substrate fluorescence possible.

### Inhibitor Phenotyping to Identify Reductases Responsible for Hydroxywarfarin Reduction

As major oxidized warfarin metabolites, *rac*-7- and 10 hydroxywarfarin attain the highest levels in patients (Locatelli, et al., 2005; Pouncey, et al., 2018), and thus, reductases involved in their metabolism may have clinical relevance. Consequently, we identified those enzymes using inhibitors relatively specific for common cytosolic reductases. Under steady state conditions established in the control experiments, reactions contained 250 µM *rac*-hydroxywarfarin or warfarin (positive control) along with either methanol alone (control) or with inhibitor (5% final methanol from carrier solvent). Reactions were conducted using three different inhibitors to selectively inhibit individual and classes of cytosolic reductases as discussed in a previous study (Barnette, et al., 2017) and review (Malátková and Wsól 2014). We employed concentrations about 5-fold above reported IC_50_ values to ensure target inhibition while minimizing off target effects. We used flufenamic acid (FFA) at a lower concentration (2 µM) to more selectively inhibit AKR1C3 and at a high concentration (10 µM) to inhibit all Aldo-keto reductase family 1C members (AKR1C). Similarly, indomethacin (IND) at lower concentration (5 µM) selectively blocks AKR1C3 activity with only limited effects on CBR1 inhibition, but at a high concentration (180 µM), indomethacin broadly inhibits all AKR1C members and Carbonyl Reductase 1 (CBR1). Lastly, we used 60 µM quercetin to target only CBR1 activity for inhibition.

## Results

### Modeling Enabled Ranking of Reduction Predictions for Hydroxywarfarin Isomers

**FIGURE 2:**
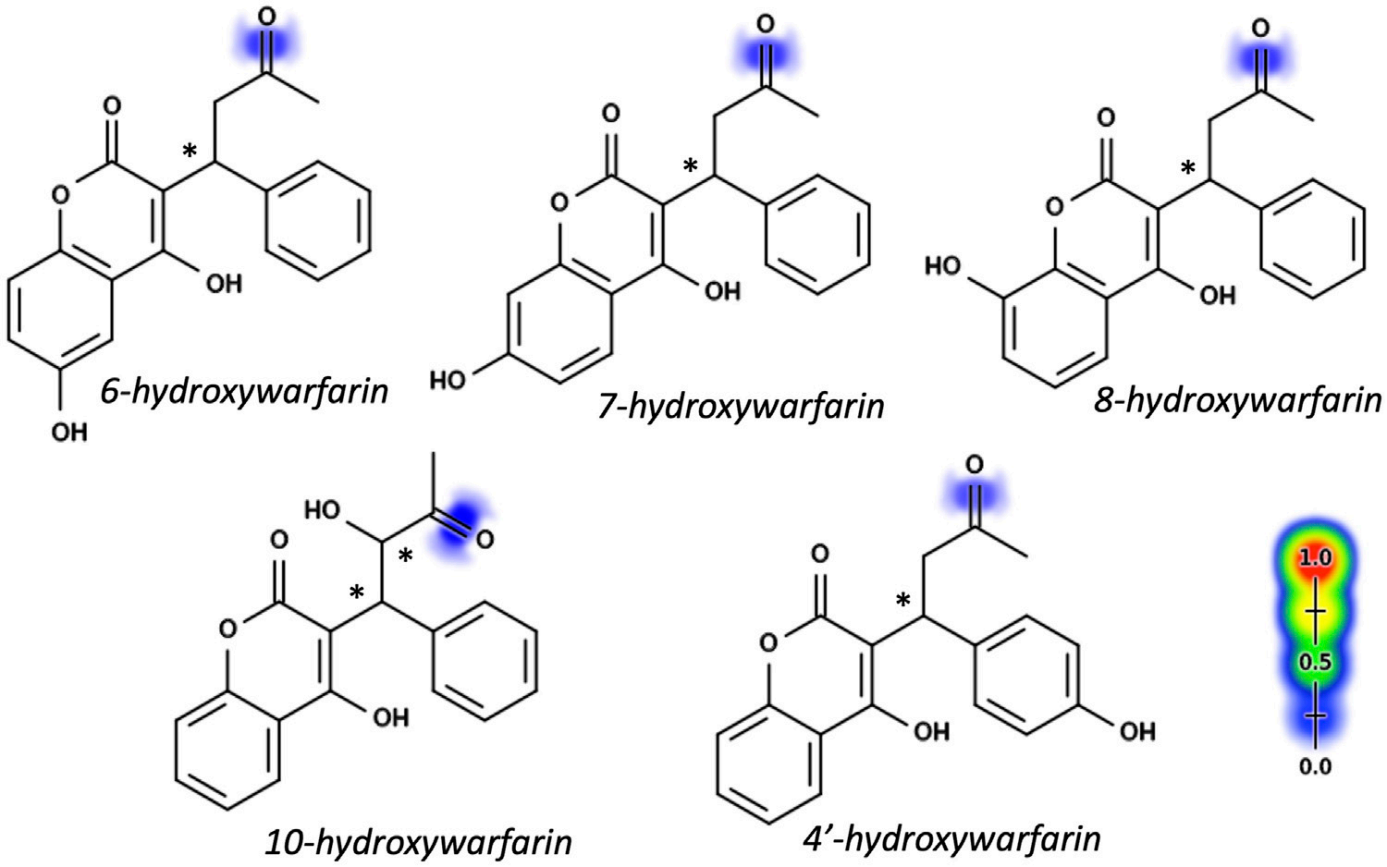
Hydroxywarfarin alcohols with reduction model predictions. Reduction predictions for hydroxywarfarins were made using the Xenosite Rainbow Model (Dang, et al., 2020). Individual bonds are scored from 0 to 1 with higher scores indicating higher likelihood of reduction and indicated pictorially with color shading from blue to red. Scores for reduction at the C11 position to form hydroxywarfarin alcohols are shown in Table 1. Asterisks denote chiral centers. All hydroxywarfarins contain a chiral center at C9 except for 10-hydroxywarfarin, which contains another at position 10.

Warfarin and the hydroxywarfarins were all predicted to undergo reduction at the C11 carbonyl bond with a moderate to low likelihood compared to the rest of the Rainbow Model training set. Figure 2 illustrates the predicted likelihood for reductive reactions, while the actual values are reported in Table 1. For comparative purposes, we normalized model predictions for hydroxywarfarins to warfarin. That strategy showed that introduction of the hydroxyl group impacted the predicted reduction of the carbonyl. When the hydroxyl group was added to the 6, 7, or 8 position on the 4-hydroxy-coumarin scaffold, there was a slight decrease in possible reductions. A significant reduction occurred upon inclusion of a 4′ hydroxyl group on the phenyl group distal from the site of reduction. By contrast, a hydroxyl group at position 10 for 10-hydroxywarfarin resulted in the highest likelihood of reduction at the C11 carbonyl group.

**TABLE 1:** Model predictions for reduction of warfarin and hydroxywarfarin isomers.

	Absolute reduction score^a^	Relative reduction score^b^
Warfarin	0.204	1
6-hydroxywarfarin	0.195	0.96
7-hydroxywarfarin	0.195	0.96
8-hydroxywarfarin	0.195	0.96
10-hydroxywarfarin	0.256	1.25
4′-hydroxywarfarin	0.176	0.86

a Output value generated by Xenosite Rainbow Model (Dang, et al., 2020) for reduction reactions.

b Relative values calculated by dividing reduction scores by that of warfarin.

### Hydroxywarfarins Underwent Reduction Into Isomeric Alcohols

**FIGURE 3:**
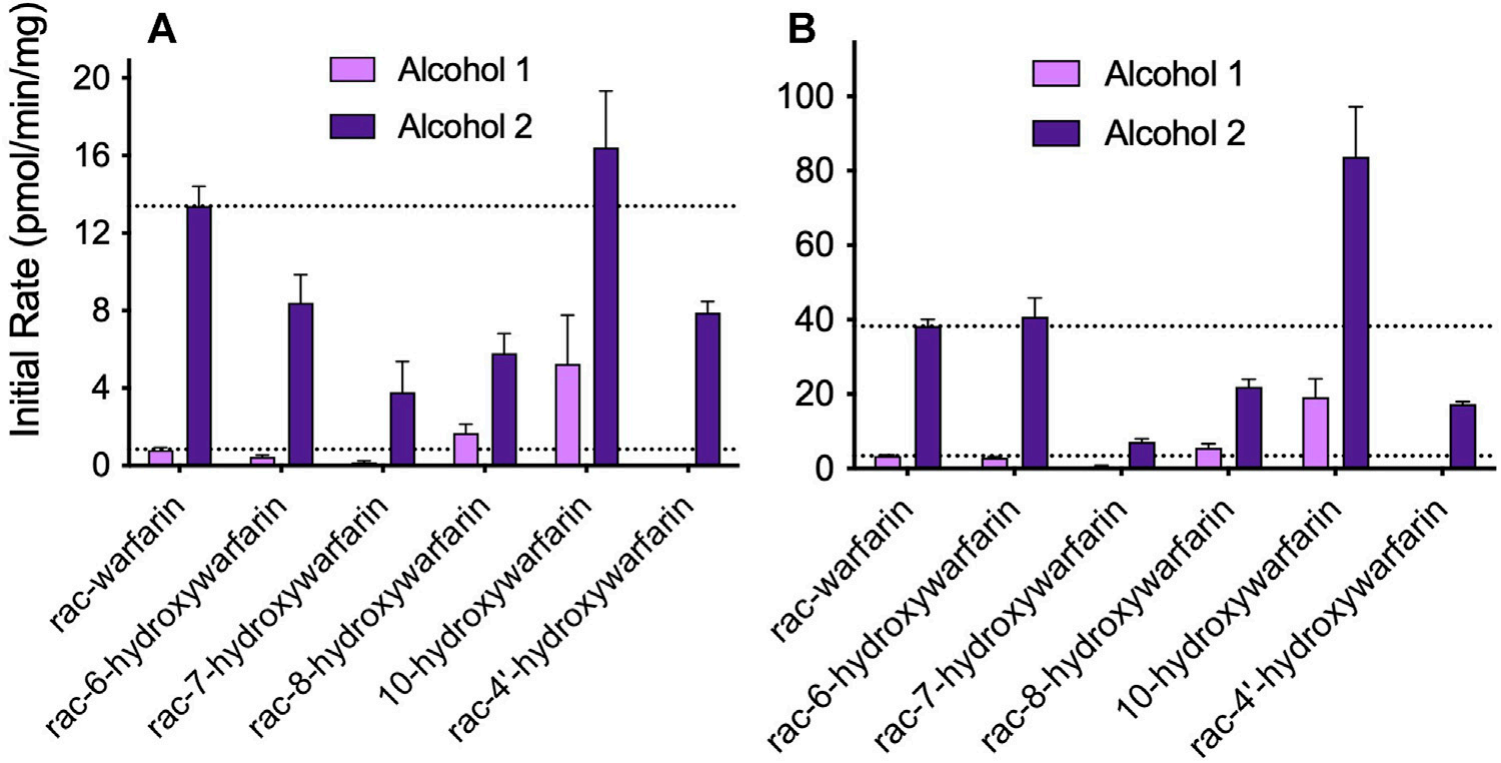
Screening hydroxywarfarins for reduction by human liver cytosol. Initial reactions were carried out with 1.0 mg/ml protein human liver cytosol in 50 mM potassium phosphate pH 7.4 with either **(A)** 100 or **(B)** 1,000 μM *rac*-warfarin (positive control) or *rac*-hydroxywarfarin. Reactions yielded alcohol 1 (minor metabolite, light purple) and alcohol 2 (major metabolite, dark purple), although the reaction for *rac*-4′-hydroxywarfarin generated only alcohol 2.

**FIGURE 4:**
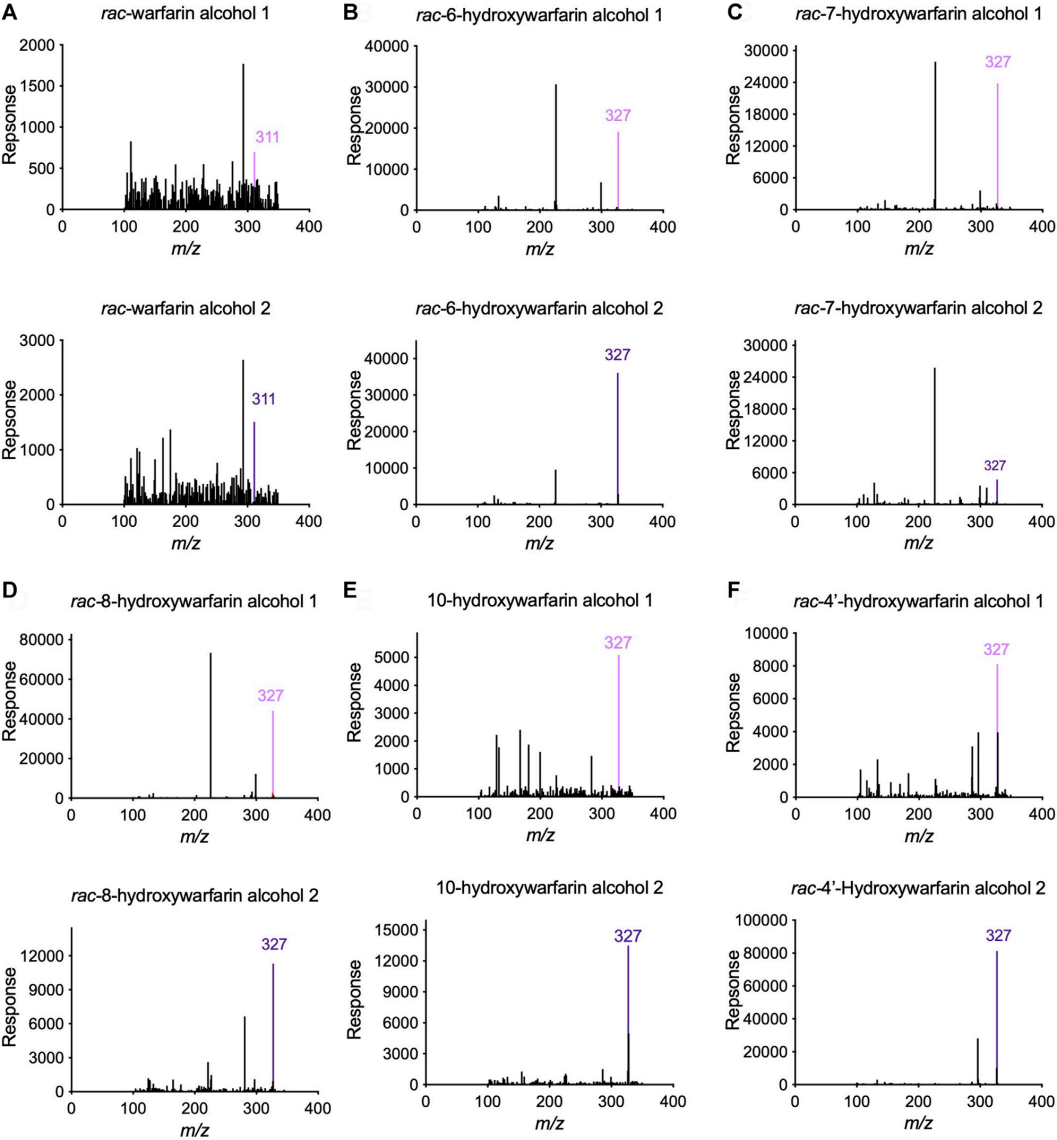
Tandem Mass Spectrometry (LC-MS/MS) analysis confirmed the predicted masses of the reduced hydroxywarfarin metabolites with reduced warfarin as a control. Parent mass scans were conducted for the metabolites from cytosolic reduction reactions for **(A)** *rac*-warfarin, **(B)** *rac*-6-hydroxywarfarin, **(C)** *rac*-7-hydroxywarfarin, **(D)** *rac*-8-hydroxywarfarin, **(E)** 10-hydroxywarfarin isomer mixture, and **(F)** *rac*-4′-hydroxywarfarin. Each panel includes the mass spectrum for alcohol 1 (top) and alcohol 2 (bottom). The colored line indicates the expected and observed 311 m*/z* for warfarin alcohol 1 (light purple) and alcohol 2 (dark purple) and the expected and observed 327 m*/z* for hydroxywarfarin alcohol 1 (light purple) and alcohol 2 (dark purple), respectively.

Initially, we screened *rac*-hydroxywarfarins for the potential for reduction by pooled human liver cytosolic fractions (HLC150) with *rac*-warfarin serving as a positive control (Figure 3). Like the parent drug, *rac*-hydroxywarfarins underwent metabolism into a minor metabolite (alcohol 1) followed by a major metabolite (alcohol 2). Unlike warfarin, the addition of a hydroxyl group impacted specificity of reduction rates and their relative values for alcohols 1 and 2. In fact, the minor alcohol 1 metabolite was not observable for *rac*-4′-hydroxywarfarin reactions. In general, *rac*-10-hydroxywarfarin underwent the highest rates of reduction for both alcohols followed by warfarin and 6-hydroxywarfarin with 7-, 8-, and 4′-hydroxywarfarin showing the lowest rates. Lastly, differences in these initial rates for *rac-*warfarin and the hydroxywarfarins were not the same at 100 and 1,000 μM, suggesting differences in the respective binding specificities, e.g., K_m_, among reactions. Subsequent LC-MS characterization provided evidence for the expected metabolite structures, namely, 311 m*/z* for warfarin alcohols (positive controls) and 327 m*/z* for hydroxywarfarin alcohols (Figure 4).

### Steady-State Reduction Kinetics Varied Among Hydroxywarfarins Compared to Warfarin

**FIGURE 5:**
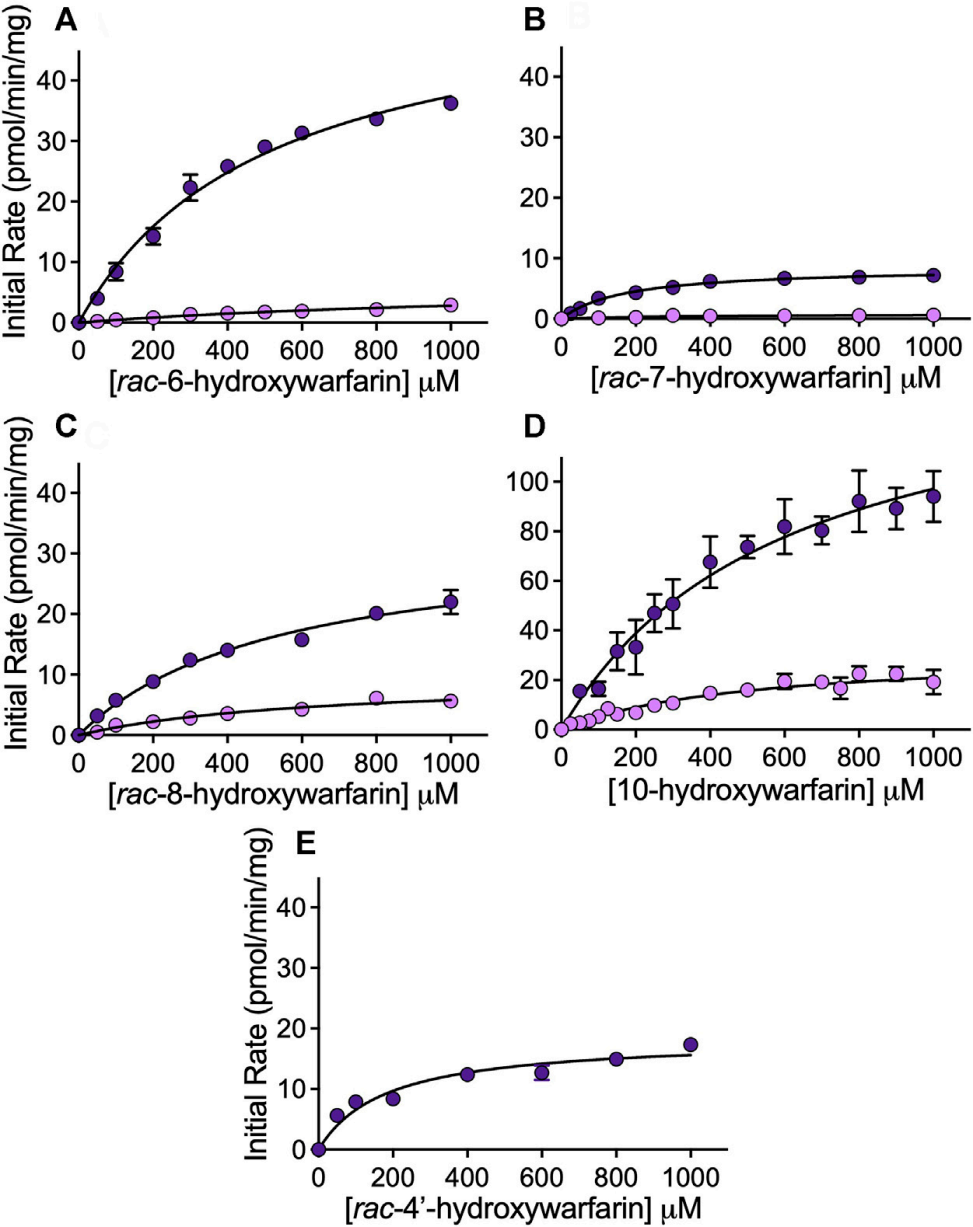
Kinetic profiles for reduced metabolites formed from hydroxywarfarin metabolism by human liver cytosol. Steady-state reactions were carried out with 1.0 mg/ml protein human liver cytosol in 50 mM potassium phosphate pH 7.4 with varying concentrations of substrate. Plots of the initial rates for alcohol 1 (light purple) and alcohol 2 (dark purple) as a function of hydroxywarfarin concentration were fit to Michaelis-Menten mechanisms for reduction of **(A)** *rac*-6-hydroxywarfarin, **(B)** *rac*-7-hydroxywarfarin, **(C)** *rac*-8-hydroxywarfarin, **(D)** 10-hydroxywarfarin isomer mixture (note difference in scaling versus other plots), and **(E)** *rac*-4′-hydroxywarfarin. The corresponding Michaelis-Menten parameters for the best fit curve are reported in Table 2. Error bars represent standard deviation of at least six replicates.

Steady-state reactions in HLC150 were conducted to determine mechanism and kinetics for reduction of hydroxywarfarin isomers compared to the warfarin parent drug. Initial reaction studies showed linearity in initial rates as a function of time and protein concentration ensuring steady-state conditions (data not shown). Figure 5 shows the resulting kinetic profiles from *rac*-hydroxywarfarin kinetic studies for minor (alcohol 1) and major (alcohol 2) metabolites. For almost all alcohols, kinetic profiles fit best to the Michaelis-Menten mechanism with the exception of alcohol 1 for rac-6-hydroxywarfarin, which did not reach saturation by 1,000 μM, so initial rates in the linear range up to 400 µM were fit to a line yielding a slope to approximate reaction specificity (V_max_/K_m_). The corresponding kinetic parameters, V_max_ and K_m_, and calculated specificities (V_max_/K_m_) for reactions are reported in Table 2. Like warfarin, the most efficient reduction pathways led to alcohol 2 and in the case of *rac*-4′-hydroxywarfarin, the minor pathway for alcohol 1 was not even measurable. In general, lower V_max_ values accompanied lower K_m_ values suggesting substrate recognition comes at the cost of substrate turnover. Overall substrate recognition was moderate to very poor as reflected in large K_m_ values, and the maximal reaction rates (V_max_) varied widely among the hydroxywarfarins and their alcohol metabolites. Moreover, the relative specificities for the pairs of alcohols for each hydroxywarfarin differed in magnitude. Taken together, these findings indicated that introduction of the hydroxyl group led to regioselectivity in metabolism but no clear patterns, when compared to warfarin. For both alcohols, *rac*-10-hydroxywarfarin had the highest specificities, and yet the order of the less specific reactions depended on the location of the hydroxyl group with *rac*-warfarin always the second most efficient.

**TABLE 2:** Michaelis-Menten kinetic parameters for reduction of warfarin and hydroxywarfarin isomers^a^.

Substrate	Kinetic constants	Alcohol 1	Alcohol 2
*rac*-warfarin^b^	Vmax (pmol/min/mg)	4.8 (3.2–6.4)	78 (57–100)
	Km (µM)	330 (48–610)	710 (350–1,100)
	Vmax/Km	0.015	0.11
rac-6-hydroxy-warfarin	Vmax (pmol/min/mg)	—	57 (52–61)
	Km (µM)	—	510 (427–593)
	Vmax/Km	0.0041^c^	0.11
rac-7-hydroxy-warfarin	Vmax (pmol/min/mg)	0.78 (0.51–1.06)	8.5 (8.1–9.0)
	Km (µM)	390 (0.64–580)	175 (140–210)
	Vmax/Km	0.0027	0.049
rac-8-hydroxy-warfarin	Vmax (pmol/min/mg)	9.54 (7.5–11.6)	33 (30–36)
	Km (µM)	644 (370–920)	540 (420–560)
	Vmax/Km	0.015	0.061
10-hydroxy-warfarin	Vmax (pmol/min/mg)	31 (27–36)	156 (130–180)
	Km (µM)	500 (340–650)	600 (430–770)
	Vmax/Km	0.063	0.26
rac-4′-hydroxy-warfarin	Vmax (pmol/min/mg)	NA	18 (17–20)
	Km (µM)	NA	180 (130–230)
	Vmax/Km	NA	0.10

a Steady-state reactions were carried out with 1.0 mg/ml protein human liver cytosol in 50 mM potassium phosphate pH 7.4 with varying concentrations of substrate.

b Barnette et al., 2017.

c Kinetic profile did not reach saturation by 1,000 μM, so initial rates in the linear range up to 400 µM were fit to a line yielding a slope to approximate reaction specificity (V_max_/K_m_).

### Multiple Reductases Were Responsible for Metabolism of 7- and 10-Hydroxywarfarin

**FIGURE 6:**
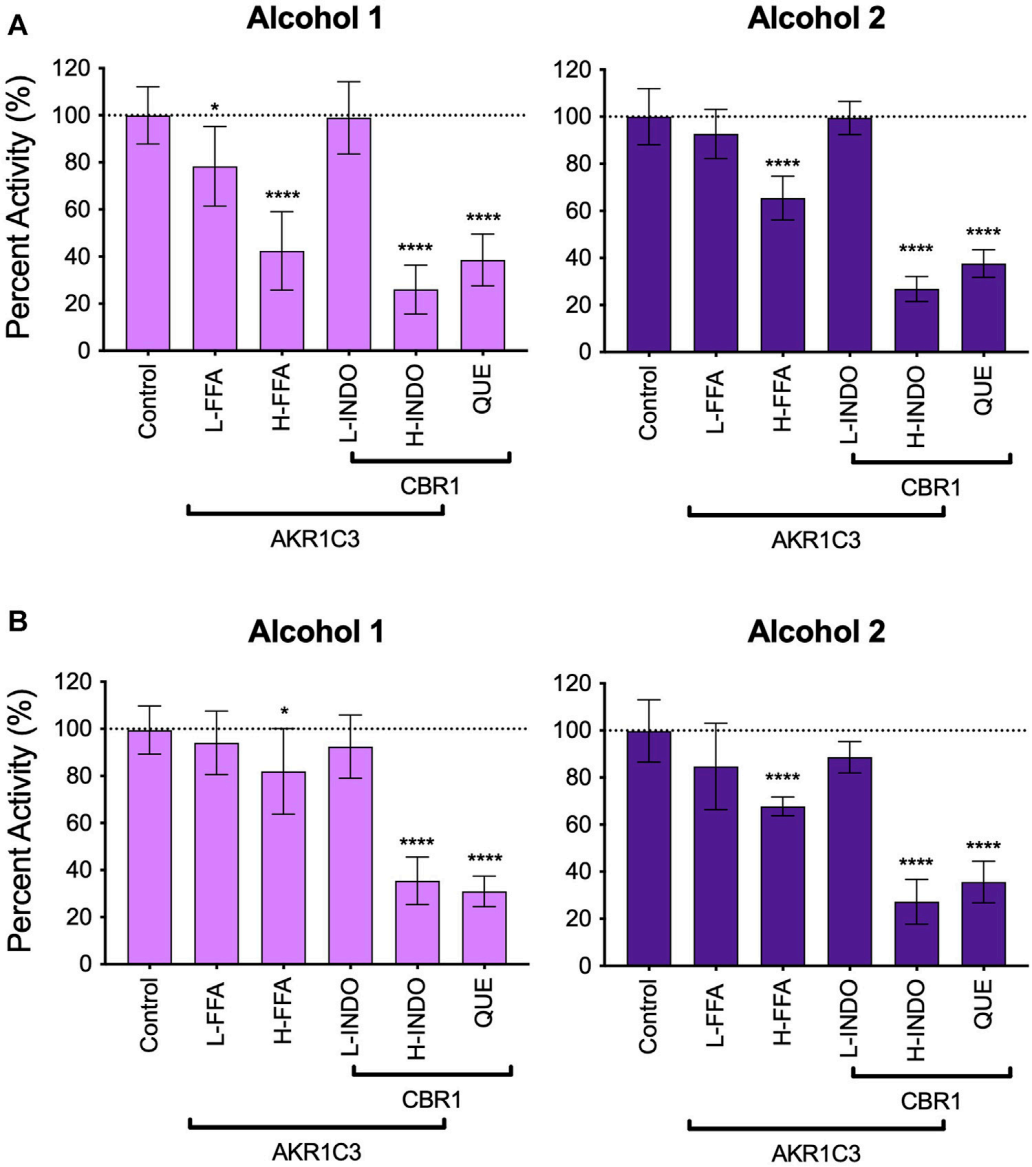
Inhibitor phenotyping for 7- and 10-hydroxywarfarin carbonyl reduction reactions in human liver cytosol. Inhibitor reactions contained 1.0 mg/ml protein human liver cytosol in 50 mM potassium phosphate pH 7.4 and 5% methanol with 250 μM substrate with and without reductase inhibitors for AKR1C3 and CBR1 for **(A)** *rac*-7-hydroxywarfarin and **(B)** 10-hydroxywarfarin. The specificities of the inhibitors are indicated by the bars in which some inhibitors and conditions were specific for either reductase while other conditions inhibited both reductases. Significance was determined using one way ANOVA statistical test. Abbreviations are as follows: L-FFA: low flufenamic acid (2 μM); H-FFA: high flufenamic acid (10 μM); L-INDO: low indomethacin (5 μM); H-INDO: high indomethacin (INDO, 180 μM); QUE: quercetin (60 μM).

Major oxidative metabolic pathways for warfarin yield primarily 7- and 10-hydroxywarfarin (Locatelli, et al., 2005; Pouncey, et al., 2018) making them more relevant to *in vivo* clearance pathways, and so we carried out phenotyping assays using reductase inhibitors to identify enzymes responsible for the reduction of those hydroxywarfarins (Figures 6A,B). The inhibition patterns for the major alcohol were similar for both substrates with about 30% inhibition by H-FFA, 60% inhibition by QUE, and 70% decrease in activity with H-INDO. Together, these results implicate significant contributions by Carbonyl reductase 1 (CBR1) and slightly less, though still significant, contribution by Aldo-Keto reductase Family 1 Member C3 (AKR1C3) toward formation of alcohol 2 for both substrates. Similarly, H-INDO and QUE decreased formation of the major alcohol from both substrate reactions by 60–70% implicating a minor role for CBR1 in the pathways. For 7-hydroxywarfarin, there was also reaction inhibition by 50% with H-FFA and 20% with L-FFA, while the inhibition was less significant inhibition for 10-hydroxywarfrin reactions. These trends suggest AKR1C3 involvement in the pathways is possibly more significant for 7-hydroxywarfarin than that for 10-hydroxywarfarin. Overall, inhibition results indicated that 7- and 10-hydroxywarfarin reduction involves mainly CBR1 with potential contributions from AKR1C3 as reported for warfarin (Barnette, et al., 2017).

### Reductases Showed Chiral Preferences in *R-* and *S*-7-Hydroxywarfarin Reductions

**FIGURE 7:**
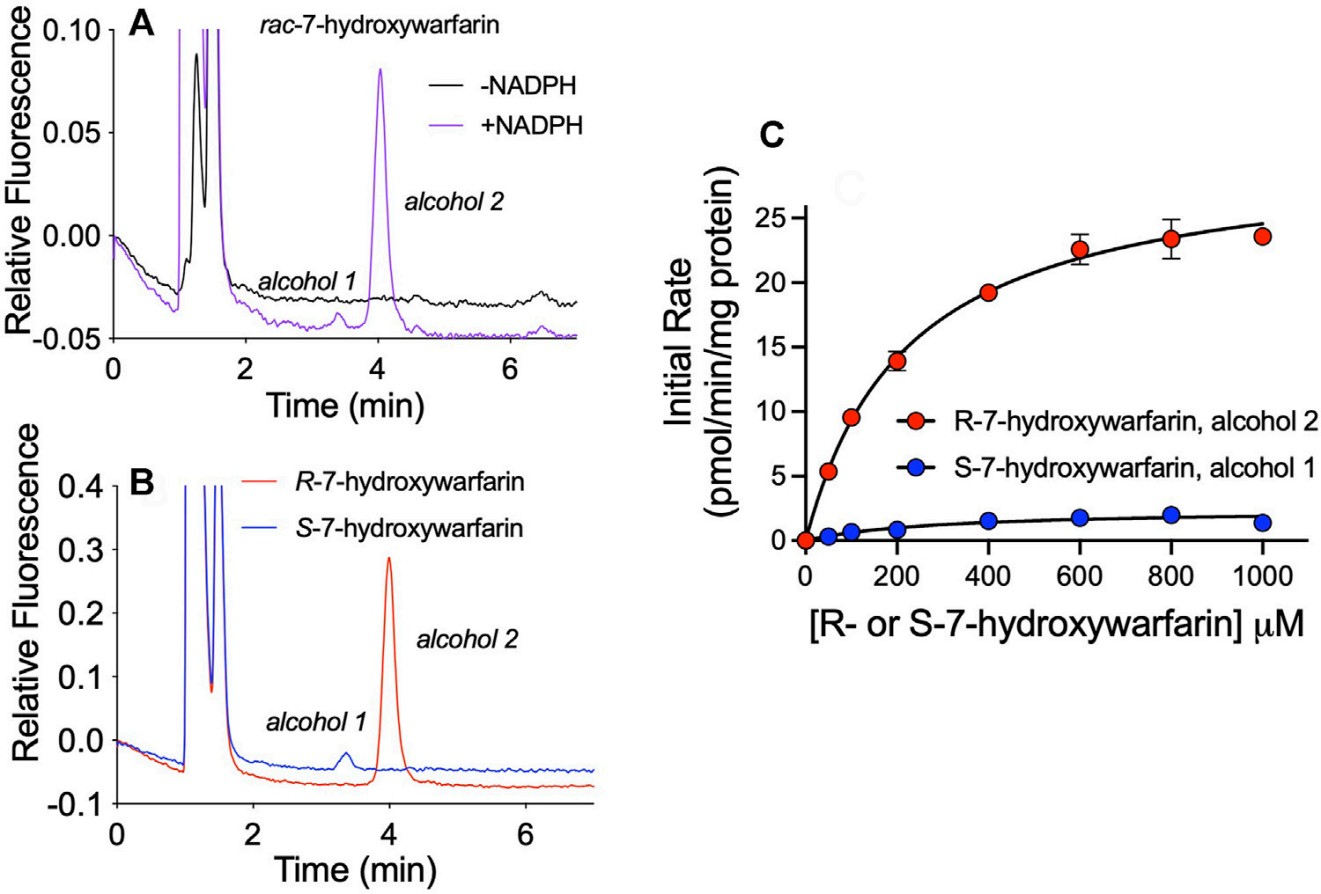
Enantioselective and enantiospecific metabolism of R- and S-7-hydroxywarfarin. An exploration of the impact of chirality on hydroxywarfarin reduction carried out with chromatographically purified 7-hydroxywarfarin enantiomers. **(A)** As a baseline for comparison, chromatographic analysis of 200 μM *rac*-7-hydroxywarfarin reduction (100 μM of each enantiomer) using 1.0 mg/ml protein human liver cytosol in 50 mM potassium phosphate pH 7.4 in the absence or presence of NADPH. **(B)** Chromatographic analysis of the reduction of 100 μM of either *R*-7-hydroxywarfarin (red) or *S*-7-hydroxywarfarin (blue) under the same reaction conditions. **(C)** Steady-state kinetics were carried out with the same reaction conditions but varying concentrations of substrate. Plots of initial rates as a function of 7-hydroxywarfarin concentration were fit to the Michaelis-Menten mechanism for *R*-7-hydroxywarfarin (red, alcohol 2) or *S*-7-hydroxywarfarin (blue, alcohol 1). The corresponding kinetic constants are reported in *Reductases Showed Chiral Preferences in R- and S-7-Hydroxywarfarin Reductions* under Results.

We sought insights on the impact of chirality toward reductase selectivity and specificity during reactions. Studies with racemic hydroxywarfarins yielded chromatographically resolved but ambiguous alcohol metabolite peaks. Individual hydroxywarfarin isomers are not commercially available; however, we had previously synthesized *R*- and *S*-7-hydroxywarfarin for studies published on their glucuronidation (Pugh, et al., 2018). In this case, we used them to assess effects of chirality on the specificity of hydroxywarfarin reduction. It was not possible to carry out similar studies with 10-hydroxywarfarin due to the presence of chiral centers at positions 9 and 10 (Jones et al., 2011) that pose a significant challenge for synthesis. As a baseline for 7-hydroxywarfarin reactions, we carried out a reaction with 200 µM *rac*-7-hydroxywarfarin resolving the minor alcohol 1 and major alcohol 2 metabolites (Figure 7A). Those reactions contained 100 µM of *R*- and *S*-7-hydroxywarfarin, so we carried out studies with the enantiomers at those concentrations (Figure 7B). When compared to the *rac*-7-hydroxywarfarin reaction, the reduction of *R*-7-hydroxywarfarin yielded a large, single peak with a retention time matching that for alcohol 2, while *S*-7-hydroxywarfarin reduction led to a small peak corresponding to the retention time for alcohol 1. Subsequent kinetic studies yielded kinetic profiles confirming those observations (Figure 7C). Both data sets fit best to the Michaelis-Menten equation. For *R*-7-hydroxywarfarin, the kinetic constants and corresponding 95% confidence intervals for alcohol 2 were V_max_ 30 pmol/min/mg protein (29–31), K_m_ 220 µM (190–260), and specificity 0.14 (V_max_/K_m_). For *S*-7-hydroxywarfarin, the values were V_max_ 2.4 pmol/min/mg protein (1.9–3.2), K_m_ 290 µM (160–550), and specificity 0.0083 (V_max_/K_m_). Taken together, these findings demonstrate that 7-hydroxywarfarin undergoes enantioselective reduction favoring to the R isomer and regiospecific product formation yielding a single metabolite for either substrate.

## Discussion

### Hydroxywarfarin Reduction Depended on Location of the Hydroxyl Group

We report the first evidence for reductases catalyzing the conversion of the C11 carbonyl of hydroxywarfarins into an alcohol as a novel pathway for their elimination. As a rapid screen for metabolism, our Rainbow metabolism model predicted that for the warfarin metabolites, reduction would increase with closer proximity of the hydroxyl group to the site of metabolism. That trend correctly ranked 10-hydroxywarfarin, as the most likely to undergo reduction followed by warfarin. Nevertheless, the pattern did not hold true for the remaining hydroxywarfarins whether considering the specificity for individual alcohol metabolites or their combination. In fact, the experimental studies did not indicate any clear trends in the specificity of reduction for the *rac*-hydroxywarfarins or 10-hydroxywarfarin that varied up to six-fold for overall specificity of reductions. *rac*-6-Hydroxywarfarin demonstrated similar metabolic kinetics as *rac*-warfarin followed by *rac*-8-hydroxywarfarin and lastly, *rac*-7-hydroxywarfarin. Moreover, the specificity of reactions for alcohol 1 and 2 differed among the hydroxywarfarin reactions. The collective findings indicate the location of the hydroxyl group significantly impacted reduction selectivity among the hydroxywarfarins, as well as the specificity for the resulting metabolites. Those effects may be due to structural discriminations of individual reductases or the collection of reductases present in cytosolic fractions.

### Reductases Carried Out Likely Enantioselective and Enantiospecific Hydroxywarfarin Metabolism

The reliance on racemic substrates for reactions masked potentially important roles for chirality in metabolic flux for individual hydroxywarfarins to the respective alcohol metabolites. For comparison, our previous work with warfarin revealed significant chiral bias in reactions (Barnette, et al., 2017). CBR1 and AKR1C3 demonstrated a higher specificity toward *R*-warfarin reduction and a preference for formation of *S* alcohols regardless of substrate chirality (Barnette, et al., 2017; Malátková and Wsól 2014). In following, reactions with *rac*-warfarin in the literature (Barnette, et al., 2017; Alshogran, et al., 2014; Malátkova, et al., 2016) then yielded predominantly a minor metabolite (alcohol 1, *9S-11S*-hydroxywarfarin) and a major metabolite (alcohol 2, *9R-11S*-hydroxywarfarin). This trend may also apply to hydroxywarfarin reactions. We explored the impact of chirality on 7-hydroxywarfarin reduction and observed many similarities to warfarin reactions (Barnette, et al., 2017). First, 7-hydroxywarfarin reductions were enantioselective toward the *R* substrate isomer. Second, reduction of either hydroxywarfarins resulted in preferentially a single alcohol despite two possibilities. This outcome indicated an enantiospecific process likely reflecting the formation of *S* alcohols based on warfarin reduction studies. The elution order pattern for warfarin alcohols matched that observed for 7-hydroxywarfarin alcohols. Moreover, both warfarin and 7-hydroxywarfarin are predominantly metabolized by CBR1 and to a lesser extent AKR1C3 that favor formation of warfarin *S* alcohols and presumably the same for 7-hydroxywarfarin. In following, 7-hydroxywarfarin reduction likely generated *9S*-7,*11S*-dihydroxywarfarin (alcohol 1, minor metabolite) and *9R*-7,*11S*-dihydroxywarfarin (alcohol 2, major metabolite). These studies suggest that the hydroxyl group at position 7 did not alter chiral bias in metabolism. If this trend extends to the other substrates in this study, then all hydroxywarfarin reductions are enantioselective toward *R* substrates and enantiospecific for *S* alcohol metabolites.

### 10-Hydroxywarfarin Reduction May be a Clinically Relevant Pathway Impacting Anticoagulation Therapy

The reduction of 10-hydroxywarfarin is the only elimination pathway that may have clinical significance. By comparison, reductive reactions for the other hydroxywarfarins were much less efficient. Moreover, 6-, 7-, 8- and 4′-hydroxywarfarins readily undergo glucuronidation (Zielinska, et al., 2007; Bratton, et al., 2012; Kim, et al., 2019; Pugh, et al., 2018) to yield glucuronides excreted in the urine (Kaminsky and Zhang 1997; Miller, Jones, et al., 2009). The effectiveness of those pathways presumably explains the very low nanomolar levels of those hydroxywarfarins in patient plasma (Haque, et al., 2014; Pouncey, et al., 2018), including *S*-7-hydroxywarfarin, a metabolite of the main pathway for *S*-warfarin elimination (Krishna Kumar et al., 2013; Rettie, et al., 1992). In contrast, 10-hydroxywarfarin is not detectable in patient urine (Miller, Jones, et al., 2009) and accumulates collectively almost up to micromolar levels in patient plasma following warfarin maintenance dosing (Haque, et al., 2014; Pouncey, et al., 2018). The resulting 10-hydroxywarfarin levels are sufficient to potentate anticoagulation directly by inhibiting VKORC1 (Haque, et al., 2014) and indirectly by inhibiting *S*-warfarin metabolism (Jones, et al., 2010c). Reported warfarin interactions with rifampicin (Kendan Alexander, 2014) and possibly nafcillin (Rulcova, et al., 2010) are consistent with those mechanisms. Both drugs induce CYP3A4 and thus, its role in metabolizing warfarin into 10-hydroxywarfarin. Clinically, the drug combinations decreased the *R*-warfarin half-life and increased the *S*-warfarin half-life resulting in warfarin sensitivity and a higher risk for bleeding. The reduction of 10-hydroxywarfarin provides a counterbalance to those outcomes. The metabolic pathway would alleviate 10-hydroxywarfarin inhibition of CYP2C9 metabolism of the pharmacologically potent *S*-warfarin and facilitate elimination of 10-hydroxywarfarin contributions to anticoagulation. In the clinic, the importance of reductases on those patient outcomes will depend on clinical factors like genetics, personal habits, and environmental factors that influence CBR1 and possibly AKR1C3 activities toward 10-hydroxywarfarin reduction (Kassner, et al., 2008).

### Limitations

The challenges in handling chirality in reactions were the main limitations of this work. The Rainbow Model lacked chiral descriptors for substrates and its application to possible metabolites. Additionally, model outputs were qualitative predictions for likelihood of a reaction to occur that did not scale to quantitative experimental kinetic mechanisms and constants. Consequently, we compared reaction specificities (V_max_/K_m_) as an approximation of model predictions for reactions to occur. The consistency of modeling and experimental results for the most efficient reactions but not the others may indicate the scaling limitations of model predictions to experimental data and/or performance of the model itself. Retraining of the model with larger, more diverse sets of reduction reactions would aid in resolving performance issues. Lastly, experimental studies relied on racemic substrates and inference for quantitating mixtures of alcohol isomers due to the absence of pure, authentic reagents. Nevertheless, interpretation of our results was possible by using our prior work with warfarin and alcohol standards along with exploratory studies with R- and S-7-hydroxywarfarin. Our results showed enantiospecificity and enantioselectivity of 7-hydroxywarfarin reduction that matched the general trend observed previously for warfarin reduction. Based on this finding, we predicted a similar trend occurs with the other hydroxywarfarin isomers.

### Concluding Remarks

Coumadin (*rac*-warfarin) is a highly efficacious drug that poses challenges to adequate dosing due to a narrow therapeutic range and sometimes unpredictable responses in patients. Critical determinants of that uncertainty are gaps in our understanding about how metabolism impacts the potency and levels of pharmacologically active parent drugs and metabolites. The studies reported herein revealed a previously unknown reduction pathway for the elimination of 10-hydroxywarfarin that may play a role in the uncertainty in patient response to warfarin therapy. Knowledge of pathways leading to 10-hydroxywarfarin by CYP3A4 and elimination by reductases provides a more complete picture of the metabolic flux for this metabolite. The resulting 10-hydroxywarfarin levels would then mediate its direct and indirect effects on the anticoagulant response that would be the foundation for important follow up clinical studies based on this research.

## Data Availability

The raw data supporting the conclusion of this article will be made available by the authors, without undue reservation.
